# Adolescent mobile phone addiction during the COVID-19 pandemic predicts subsequent suicide risk: a two-wave longitudinal study

**DOI:** 10.1186/s12889-022-13931-1

**Published:** 2022-08-12

**Authors:** Gangqin Li, Aldo Alberto Conti, Changjian Qiu, Wanjie Tang

**Affiliations:** 1 Department of Forensic Psychiatry, West China School of Basic Medical Sciences & Forensic Medicine, Sichuan University, Chengdu, Sichuan China; 2 Department of Child and Adolescent Psychiatry, Institute of Psychiatry, Psychology & Neuroscience, King’s College London, London, UK; 3 Mental Health Centre, West China Hospital, Sichuan University, Chengdu, China

**Keywords:** COVID-19, Suicidality, Depression, Daytime sleepiness, Mobile phone addiction

## Abstract

Both the rate of mobile phone addiction and suicidality among adolescents have increased during the pandemic lockdown. However, the relationship between mobile phone addiction and suicide risk and the underlying psychological mechanisms remains unknown. This study examined the associations between mobile phone addiction in adolescents during the first month of lockdown and the suicide risk in the subsequent five months. A two-wave short-term longitudinal web-based survey was conducted on 1609 senior high school students (mean age = 16.53 years, SD = 0.97 years; 63.5% female). At Time 1 (T1), the severity of mobile phone addiction and basic demographic information was collected from Feb 24 to 28, 2020 in Sichuan Province, China (at the pandemic’s peak). Five months later, between July 11 and July 23 (Time 2, T2), mobile phone addiction, daytime sleepiness, depression, and suicidality were measured within the past five months. The regression analysis revealed that mobile phone addiction during quarantine directly predicted suicidality within the next five months, even after controlling for the effect of depression and daytime sleepiness. Meanwhile, mobile phone addiction at T1 also indirectly predicted suicidality at T2, with depression and daytime sleepiness mediating this association. Programs targeting improvement of daytime sleepiness and depressive symptoms may be particularly effective in reducing suicide risk among adolescents with mobile phone addiction.

## Introduction

COVID-19 emerged in late 2019 and forced many countries to implement quarantine measures, such as lockdowns, home confinement, self-isolation, and social distancing [1], which raise public health concerns [2, 3]. Because school-aged children had to take online classes from home during the pandemic, there was a significant increase in their use of mobile phones [4, 5], which led to the rise in mobile phone addiction [6–8]. It has also been found that pandemic lockdown measures resulted in a rise in negative emotions in adolescents, which manifested as sleep problems [9], anxiety, depression, and suicidality [10–14]. Indeed, youth suicide risk has become a significant global health concern during the pandemic [15, 16]. However, neither the connection between mobile phone addiction and the increased suicide risk during the pandemic, nor the associated psychosocial mechanisms, have been adequately explored [17, 18]. Therefore, to fully understand the underlying mechanisms in the relationship between adolescent mobile phone addiction and suicidality during the COVID-19 pandemic, there has been a call for more profound investigative research [19–22].

### COVID-19 and suicidality

Many studies have investigated suicidality during the COVID-19 pandemic. For example, Every-Palmer et al. (2020) surveyed 2010 New Zealand adults in April 2020 and found that 6% had suicidal ideation and 2% had attempted suicide [23]. Iob et al. (2020) reported that 18% (*n* = 7984) of 44,775 adults in the UK had experienced thoughts of suicide or self-harm, and 5% (*n* = 2174) had harmed themselves at least once in the first month of the COVID-19 lockdown. Czeisler et al. (2020) surveyed 5412 United States adults and found that 10.7% had seriously considered suicide in the 30 days before completing the survey [24]. Wang et al. (2020) reported that 18.04% of 2031 undergraduate and graduate students in the United States had experienced suicidal ideation during the COVID-19 pandemic, and Sun et al. (2021) found that 19.56% of Chinese university students had had suicidal ideation during the COVID-19 quarantine. However, a study in Spain did not find any significant change in the overall suicidal ideation prevalence before and after the COVID-19 outbreak [25],. Similarly, Isumi et al. (2020) found that the suicide rates in children and adolescents under 20 during the school closures from March to May 2020 in Japan did not change significantly compared with the same period in 2018 and 2019.

Although many studies have investigated the rate of suicidal ideation or behaviors during the COVID-19 crisis, several research gaps need to be addressed: 1) the majority of the studies have only reported the rate of suicidal ideation, attempts, or behaviors, but few studies have investigated the underlying suicidality mechanisms; 2) most previous studies have been cross-sectional rather than longitudinal; however, as the pandemic psychological sequelae may persist for months and years to come and peak later than the actual pandemic [26], longitudinal or follow up studies are needed; 3) most studies on pandemic related suicidality have been conducted on adults, yet adolescents are vulnerable to negative emotions and may be particularly disposed to suicidality [27, 28], therefore, longitudinal studies on suicidality and the related mechanisms in adolescents are needed.

### Mobile phone addiction and suicidality

Mobile phone addiction is defined as the inability to regulate personal mobile phone use, which can eventually lead to symptoms similar to substance abuse disorders, such as compulsive use, withdrawal, cravings, loss of control, and mood dysregulation [29]. A significant increase in mobile phone use and addiction has been found in many COVID-19 studies. For example, Zhang et al. (2021) found an elevated risk of problematic mobile phone use in Chinese adults during the COVID-19 pandemic, with the prevalence being as high as 43.3%. Saadeh et al. (2021) reported a 62.4% mobile phone addiction prevalence in 6154 Jordanian undergraduates during the COVID-19 quarantine, and Caponnetto et al. (2021) and Serra et al. (2021) both found a growth in pathological cell phone use during the Italian COVID-19 lockdown.

Several studies have also found that mobile phone addiction could result in several physical and mental health issues, such as dry eyes, migraine headaches, sleep disorders, intellectual impairment, depression, and anxiety [30–32]. However, only a few studies have investigated the associations between mobile phone addiction and suicidality. For example, Chen et al. (2020) found that high mobile phone use intensity directly predicted suicide-related behaviors in Chinese adolescents and that depression mediated this relationship [33]. Ismail et al.(2020) found that smartphone addiction was positively associated with suicidality in Malaysian college students; however, no predictive effects were identified in this association [34].

### Pathways from mobile phone addiction to suicidality

It has been found that mobile phone addiction can lead to adverse psychological sequelae, with depression and sleep disturbance being the most commonly reported [35–37]. For example, Lemola et al. (2015) found excessive mobile phone use at night to cause sleep difficulties, depression, and stress in adolescents [38]. Demirci et al. (2015) reported positive correlations between mobile phone addiction scale scores, depression levels, and sleep quality scores [39]. Thomee et al.’s (2011) longitudinal study revealed an association between high-frequency mobile phone use, sleep disturbances, and depression at a one-year follow-up on 4156 young adults [40]. Elhai et al.(2017) conducted a literature review and found that nine of 10 included studies reported at least medium effect size associations between problematic smartphone use and the depression severity [41]. Other cross-sectional studies revealed correlations between high levels of mobile phone addiction severity and poor sleep quality [42–44].

In addition to the association with mobile phone addiction, depression and sleep disorders have also been associated with suicidality [45–47]. For example, Bernert et al.’s (2015) systematic review concluded preliminary, converging evidence that sleep disturbances were an empirical risk factor for suicidal behaviors. Liu et al. (2004) found that sleeping less than eight hours and frequent nightmares were significantly associated with an increased risk of suicide attempts in adolescents. Wang et al. (2021) found that sleep disturbances could prospectively predict the development and persistence of suicidal ideation. Daytime sleepiness, defined as an inability to maintain wakefulness and alertness during the day [48], is one of the most common sleep disturbance sequelae [49], which is most directly associated with daytime dysfunction. Notably, studies have proposed that daytime sleepiness may mediate the association between mobile phone addiction and PTSD in adolescents during COVID-19 (Hu, Wang, et al., 2021). It is also possible for daytime sleepiness to play a mediating role between mobile phone addiction and suicidality [50, 51].

Therefore, as mobile phone addiction can result in depression, sleep disturbances, and daytime sleepiness, which in turn may increase suicidality, it is reasonable to assume that depression and daytime sleepiness could mediate between mobile phone addiction and suicidality. However, the above evidence illustrates that only a few studies have explored the underlying mediating mechanisms between mobile phone addiction and suicidality in a longitudinal design. For instance, a Chinese study has shown that depression mediates the relationship between mobile phone addiction and suicidal behavior in adolescents in a cross-sectional study [52]. Another study found that insomnia and depression mediated the relationship between internet gaming addiction and suicidal ideation [53]. As prior studies have been primarily cross-sectional, there is a need to conduct longitudinal studies investigating these associations, and the underlying mechanisms, in adolescents during the COVID-19pandemic.

### The present study

Research into the complex interactions and associations between mobile phone addiction, suicidality, depression, and daytime sleepiness could provide a more comprehensive understanding of the potential suicidality mechanisms. Therefore, this study was a two-wave longitudinal study aimed at investigating: (a) the severity of mobile phone addiction in Chinese adolescent students during the COVID-19 quarantine (Time 1, T1), and (b) the suicide risk five months later (Time 2, T2) when the pandemic was in remission in China. Specifically, we tested the following hypotheses: (1) That mobile phone addiction (T1) would be positively correlated with suicidality (T2); (2) That mobile phone addiction (T1) would predict suicidality (T2); (3) That depression and daytime sleepiness would mediate the link between mobile phone addiction (T1) and suicidality (T2). These findings may advance the understanding of how and when mobile phone addiction causes suicidality and how to protect adolescents from the unfavorable impacts of excessive mobile phone use.

## Methods

### Participants and procedures

Students from two senior high schools (12 ~ 18 years old, those over 18 are excluded) in Sichuan province, China, were invited to participate in an online survey for basic demographic information on mobile phone addiction from February 24 to 28, 2020 (T1, about one month after the national lockdown in China that began on January 21, 2020). Five months later, between July 11 and July 23 (Time 2, T2), mobile phone addiction, daytime sleepiness, depression, and suicidality in the past five months were measured. In Sichuan Province, the state lockdown was lifted at the end of March 2020, and students returned to school in early April. Therefore, the COVID-19 pandemic was considered in remission at Time 2.

This study was approved by the Ethics Committee of the Sichuan Psychological Society (NO. 2020_12). Informed consent was given online by participants and their statutory guardians. This survey was part of the Surveys on the Behavior and Psychological Health Project affected by COVID-19.

### Measures

Measures included demographic variables, pandemic exposure questionnaires, and four psychopathological conditions, including mobile phone addiction, depressive symptoms, daytime sleepiness, and suicidal ideation or attempts. At T1, we measured mainly what happened in the past month, within the first month during the lockdown. At T2, we measured mainly what occurred in the past five months, that is, within the five months between T1 and T2.

#### Demographic variables and COVID-19 related exposure

Demographic information on age, gender, and grade level was collected. COVID-19 exposure was evaluated with three yes/no questions derived from previous traumatic studies [54, 55], whether a friend or relative had been infected with COVID-19; whether the participants lived in a community in which someone was infected; whether the participants had a friend or relative who had died of COVID-19.

#### Mobile Phone Addiction Index (MPAI)

Mobile phone addiction at T1 was assessed using the Chinese version of the Mobile phone Addiction Index (MPAI) [56], which was a self-report questionnaire with 17 items under four subscales: losing control and receiving complaints; anxiety, and cravings; withdrawal/escape; and productivity loss. At T1, MPAI was measured within the first month of the lockdown. The participants were required to rate each item on a five-point Likert scale ranging from 1 = never to 5 = very often. The total MPAI score ranged from 17 to 85, with higher scores indicating a higher intensity of mobile phone use and 51 or above showing mobile phone addiction [57]. In the present study, the Cronbach’s a is 0.92 at T1 and 0.94 for the T2.

#### Daytime sleepiness

The participants’ daytime sleepiness at T2 was evaluated using the Chinese Adolescent Daytime Sleepiness Scale (CADSS) [58] for the past five months between T1 and T2. The CADSS comprised seven items; each scored on a five-point Likert scale. Higher scores indicate more severe daytime sleepiness (Range = 7–35), and a score greater than 16 indicates excessive daytime sleepiness. In the current study, Cronbach’s α was 0.89.

#### Depression

Depression at T2 was assessed using the abbreviated Kutcher Adolescent Depression Scale (KADS) in the past five months, with six items and a self-report scale to diagnose adolescent depression and its severity [59]. This scale has been used on Chinese children and adolescents and has shown good internal and test–retest reliability [60]. The total KADS score ranged from 0 to 18, with scores ≥ 6 indicating possible depression. The Cronbach’s α in the current study was 0.91.

#### Suicidality

At T2, suicidality in the past five months between T1 and T2 was measured using the modified Chinese version of the Suicidal Behaviors Questionnaire-Revised (SBQ-R) [61], which is a brief suicidality self-reported scale that has four questions, such as “Have you ever thought about or attempted to kill yourself in the past five months?” marked on a Likert scale on the frequency of suicidal ideation or attempts, with the total score ranging from 3 to 18 and scores ≥ 7 indicating a non-clinically significant risk of suicide. This questionnaire's good psychometric properties have been reported in previous studies on Chinese adolescents and young adults [62]. In the present study, Cronbach’s α was 0.76.

### Data analysis

Pearson’s correlation analysis was used to examine the clinical variable associations; To identify whether and how much variance mobile phone addiction at T1 were independent predictors of subsequent suicidal risk, stepwise and enter regression analyses were carried out while controlling for demographic and COVID-19 exposure factors, daytime sleepiness, and depressive symptoms step by step. The mediation analysis adopts PROCESS model 6 [63], which allows four variables to exist in a chain effect to examine the mediating effects of daytime sleepiness/depression on the associations between mobile phone addiction and suicidality. The indirect effects and 95% bootstrap confidence intervals (CI) were calculated on 5000 bootstrapped samples as conducting the bootstrap resampling method requires at least 2000 replications [64] and more bootstrapped samples improve estimation [65]. The statistical analyses were conducted using SPSS version 22.0.

## Results

### Demographic information and COVID-19 exposure

Two thousand three hundred ninety-nine participants were initially surveyed from the two senior high schools; 1609 (73.6%) completed the T1 and T2 surveys. The mean age for these 1609 adolescents (range 12 ~ 18 years old) was 16.53 (SD = 0.97) years, 1021 (63.5%) of whom were female. Three hundred and twenty-two (20.0%) students reported suicidal ideation or suicide attempts at T2, 207(64.3%) females. The detailed sample characteristics are presented in Table 1. Suicidality stratified by demographic and exposure variables were displayed in Table 2.

**Table 1:** Demographic and exposure variables in a sample of Chinese adolescents (*N* = 1609)

Variables	N	%
Total	1609	100
Gender		
Male	588	36.5
Female	1021	63.5
Age(yr)		
≤ 15	243	15.1
16	526	32.7
17	552	34.3
18	288	17.9
Grade		
10	485	30.1
11	692	43.0
12	432	26.9
Only-child status		
Yes	397	24.7
No	1212	75.3
Someone in the community is infected		
Yes	92	5.7
No	1517	94.3
A relative or friend is infected		
Yes	13	0.8
No	1596	99.2
A relative or friend died from the infection		
Yes	3	0.2
No	1606	99.8
Smoker		
Yes	31	19.3
No	1578	80.7
Drinker		
Yes	93	5.8
No	1516	94.2

**Table 2:** Suicidality stratified by demographic and exposure variables (*N* = 1609)

Variables	Suicidality (n)	Prevalence(%)	χ^2^
Total	*n *= 322	20.0	
Gender			0.12
Male	115	19.6	
Female	207	20.3	
Age(yr)			3.44
≤ 15	38	15.6	
16	110	20.9	
17	115	20.8	
18	59	20.5	
Grade			2.68
10	85	17.5	
11	146	21.1	
12	91	21.1	
Only-child status			0.05
Yes	81	20.4	
No	241	19.9	
Someone in the community is infected			0.48
Yes	21	22.8	
No	301	19.8	
A relative or friend is infected			1.24
Yes	1	7.7	
No	321	20.1	
A relative or friend died from the infection			0.75
Yes	0	0	
No	322	20.0	
Smoker			2.96**
Yes	10	32.3	
No	312	19.8	
Drinker			0.41
Yes	21	22.6	
No	301	19.9	

^**^p < 0.01

### Correlations between the main variables

Mobile phone addiction at T1 was significantly correlated with daytime sleepiness (*r* = 0.316), depression (*r* = 0.312), and suicidality (*r* = 0.289) at T2. Meanwhile, suicidality at T2 was significantly correlated with depression at T2 (*r* = 0.469) and daytime sleepiness at T2 was also significantly correlated with suicidality at T2 (*r* = 0.311). Details are present in Table 3. The correlations between these four variables indicated that subsequent regression was needed.

**Table 3:** Correlations of main study variables (*N* = 1609)

Variable	M	SD	1	2	3	4
1. MPA_T1	41.60	12.59	1			
2. DS_T2	13.45	5.80	0.316**	1		
3. Depression_T2	4.22	3.73	0.312**	0.364**	1	
4. Suicidality_T2	4.68	2.88	0.289**	0.311**	0.469**	1

^**^p < 0.01

*MPA_T1* Time 1 smartphone addiction score, *DS_T2* Time 2 daytime sleepiness score, *Depression_T2* Time 2 depression score, *Suicidality_T2* Time 2 suicidality score

### The chain mediation model

**Fig. 1:**
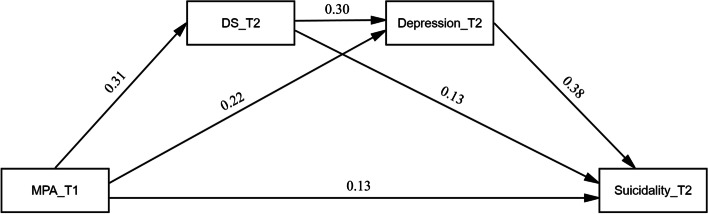
The standardized path coefficients in chain mediation model. MPA_T1: Time 1 smartphone addiction score; DS_T2: Time 2 daytime sleepiness score; Depression_T2: Time 2 depression score; Suicidality_T2: Time 2 suicidality score

The pathways between the main variables are shown in Table 4 and Fig. 1. The total standardized effect of the three variables on the outcome variable of suicidality was 0.29 (95% CI [0.24, 0.34], F = 146.12, *p* < 0.001). Mobile phone addiction at T1 was significantly directly related to suicidality at T2 (β = 0.13; 95%CI: 0.08–0.17; *p* < 0.001). Additionally, Mobile phone addiction had indirect effects on suicidality at T2 through daytime sleepiness (β = 0.04, SE = 0.01, 95%CI = 0.02–0.06), and depression (β = 0.08, SE = 0.01, 95%CI = 0.06–0.11) respectively. Furthermore, mediation paths of mobile phone addiction → daytime sleepiness → depression → suicidality (β = 0.04, SE = 0.01, 95%CI = 0.03–0.05) was also identified by the mediation analyses.

**Table 4:** Results of the Chain Mediating Effect with daytime sleepiness and depression as mediators between mobile phone addiction and suicidality

Path	Effect	SE	p	95% CI
MPA_T1(X) → DS_T2(M1) → Depression_T2(M2) → Suicidality_T2(Y)				
Total effect of X on Y	0.289	0.024	< 0.001	0.242–0.336
Direct effect of X on Y	0.129	0.023	< 0.001	0.083–0.174
Indirect effect of X on Y(Total)	0.160	0.014	-	0.133–0.189
Indirect effect of X on Y(Via M1)	0.042	0.010	-	0.023–0.062
Indirect effect of X on Y(Via M2)	0.083	0.012	-	0.059–0.110
Indirect effect of X on Y(Via M1 → M2)	0.036	0.006	-	0.026–0.047
X → M1	0.316	0.026	< 0.001	0.265–0.367
M1 → Y	0.131	0.030	< 0.001	0.073–0.189
X → M2	0.218	0.027	< 0.001	0.162–0.271
M2 → Y	0.381	0.035	< 0.001	0.316–0.451
M1 → M2	0.295	0.029	< 0.001	0.238–0.353

Note. *MPA* Mobile phone addiction, *DS* Daytime sleepiness

### Stepwise linear regression analysis results

In our data, earlier mobile phone addiction could directly predict subsequent suicidal risk when controlling for age, gender, exposures, depressive symptoms and daytime sleepiness (β = 0.13, t = 5.60, ΔR^2^ = 0.015, *p* < 0.001) (see Table 5). Depression_T2 and daytime sleepiness_T2 were also significant risk factors for suicidality_T2 in the regression model, with depression showing the most statistical significance.

**Table 5:** Stepwise regression of suicidal risk with age, gender, COVID-19 related exposure, daytime sleepiness, depressive symptoms, and MPAI in adolescents (*N*=1609)

Independent variable	Step 1: Adjusted *R*^2^=0.001	Step 2: Adjusted *R*^2^= 0.003	Step 3: Adjusted *R*^2^= 0.244	Step 4: Adjusted *R*^2^= 0.259
	Beta	Beta	Beta	Beta
Age	0.019	0.019	0.021	0.021
Gender	0.026	0.025	0.006	0.004
Someone in the community is infected		0.010	0.008	0.011
A relative or friend is infected		-0.037	-0.030	-0.035
A relative or friend died from the infection		-0.025	-0.018	-0.019
Daytime sleepiness_T2			0.410***	0.381***
Depression_T2			0.160***	0.130***
MPAI_T1				0.130***

*Abbreviations*: *MPA_T1* Time1 mobile phone addiction score, *Daytime sleepiness _T2* Time 2 daytime sleepiness score, *Depression_T2* Time 2 depression score

***p < 0.001

## Discussion

This study is one of the few longitudinal studies that have examined the associations between mobile phone addiction and suicidality in adolescents during COVID-19. It was found that mobile phone addiction during the COVID-19 quarantine period could directly predict suicidality in the subsequent five months even after controlling for the effect of depression and daytime sleepiness. In addition, mediation analysis showed that mobile phone addiction during the COVID-19 quarantine period could indirectly predict suicidality in the following five months, with depression and daytime sleepiness mediating in this association.

### Mobile phone addiction predicts suicidality regardless of mental health problems and other covariables

The regression analysis revealed that mobile phone addiction during the COVID-19 quarantine period could directly predict suicidality independent of covariables such as mental health and psychosocial factors in the subsequent five months. Several previous correlation studies supported this result. For example, Steinbüchel reported that 48.3% of the patients with Internet addiction (with and without comorbidity) exhibited significantly more often suicidal symptoms as compared to healthy controls (3.5%) [66]. Two nationally representative surveys of U.S. adolescents (*N* = 506,820) reported that among those who used electronic devices five or more hours a day, 48% had at least one suicide-related outcome [67]. Furthermore, the meta-analysis also found an increased risk of suicidal behavior in adolescents with internet addiction [68].

Our results confirmed and extended a previous cross-sectional study that internet overuse by children and adolescents in the context of COVID-19 could elevate their risks for self-injured behavior [69]. Actually, both the rate of phone addiction [70] and the risk of suicide [71, 72] among teenagers have risen dramatically during the pandemic although few studies, especially longitudinal design, confirmed the relationship between mobile phone overuse and subsequent suicide risk. Therefore, our study has important implications for considering the important role of mobile phone addiction when enacting suicide prevention programs for adolescents.

Mobile phone addiction has been related to various negative psychosocial consequences, which may contribute to subsequent suicidality. Schutten et al. reported that heavy media addiction was at risk for problematic behaviors such as substance abuse, overeating, problematic gambling, and poor financial management [73]. These unhealthy lifestyles would result in poor life performance, daily life function, social relationships, and academic or occupational achievements [74–76]. Furthermore, heavy screen-addicted adolescents were also found to have less social support and attachment with family and peers[77–79]. Poor social support would increase the risk of further isolation and loneliness, leading to suicidality [80–82].

In addition to the negative psychosocial consequences, mobile phones and internet addiction has been also associated to structural and functional abnormalities in brain areas related to cognitive control and emotional regulation. For example, Cheng and Liu found that internet addiction subjects had decreased negative functional connectivity (FC) between the dorsolateral prefrontal cortex (DLPFC) and amygdala [83], which is responsible for emotion-cognition interactions [84]. Turel et al. showed that Facebook users with addiction-like symptoms have a hyperactive amygdala-striatal system [85]. He et al. found that social network site addiction is associated with a more impulsive brain system, manifested through reduced gray matter volumes in the amygdala bilaterally [86]. Similarly, Dong et al. found that males with Internet addiction showed significantly greater ‘Stroop effect’-related activity in the anterior and posterior cingulate cortices than healthy males, indicating diminished efficiency of response-inhibition processes [87].

### Depression was a mediator between mobile phone addiction and suicidality

The mediation analysis identified a pathway from mobile phone addiction to depression to suicidality, which confirmed the mediating role of depression in the association between mobile phone addiction and suicidality. This result was consistent with a previous cross-sectional study reporting that depression mediated the relationship between high-intensity mobile phone use and suicide-related behaviors in Chinese adolescents [33]. Depression has been one of the most commonly reported adverse psychological sequelae associated with mobile phone addiction, which is characterized by depressive moods, such as adolescent feelings of irritability or emptiness, diminished interest, loss of pleasure, feelings of hopelessness and worthlessness, changes in appetite or sleep, and reduced energy or fatigue.

Several reasons have been proposed for the pathogenesis of addiction-related depression. First, mobile phone addiction can lead to work or school performance impairments [88], which could induce criticism, blame, or arguments from teachers and family members, which could, in turn, lead to impaired social relationships, low self-esteem, and negative emotions, such as depression [29]. This may result in a more compulsive mobile phone use to escape these real-world problems and alleviate any negative emotions [30, 89]. Therefore, there seems to be a vicious cycle between mobile phone addiction and depression and subsequent suicidality risk [41]. As depression has long been recognized as one of the most critical risk factors for suicide prediction [45, 47], more attention needs to be paid to its presence when screening people with mobile phone addiction and the intervening suicidality. As it may be challenging to evaluate the severity of the negative influence of mobile phone addiction and when and how to implement an intervention, the presence of depression could be a valuable warning sign; that is, greater attention should be focused on adolescent mobile phone addicts who also have depressive symptoms.

### Daytime sleepiness was a mediator between mobile phone addiction and suicidality

The mediation analysis indicated that mobile phone addiction was associated with suicidality, and daytime sleepiness mediated this association. Daytime sleepiness, defined as the inability to maintain wakefulness and alertness during the day [48], is one of the most common sleep disturbance sequelae [49]. Adolescents with mobile phone addiction tend to spend more time on their mobile phones at night, which often leads to decreased sleep duration and circadian rhythm disturbances from the mobile phone’s light and electromagnetic fields that can negatively influence serum melatonin and cerebral blood flow [90–92]. Extensive mobile phone use can also result in frequent headaches, tension, fatigue, and vertigo [93], resulting in sleep disturbances.

Sleep disturbance has also been identified as a risk factor for suicidality [94]. For example, a recent two-year follow-up study found that shorter sleep duration at the baseline was associated with an increased suicidality risk in school-aged boys [95], and another longitudinal analysis found that short sleep durations predicted the onset or persistence of suicidal ideation [96]. One possible mechanism for this association may be that sleep deprivation and circadian disturbances compromise frontal lobe/executive functions, diminish problem-solving abilities, and increase impulsive behavior, increasing the likelihood of suicide [97]. Insufficient sleep can also lead to mood regulation impairments and improve suicide ideation [98] and has been associated with hypothalamus/hypothalamic–pituitary–adrenal (HPA) axis dysregulation, which has been linked to depression and suicidality risk [99–101].

Daytime sleepiness has been strongly associated with sleep disturbance, which is more directly associated with daytime dysfunction compared to sleep disturbance. This study identified the mediating role of daytime sleepiness on the association between mobile phone addiction and suicidality. Although these are only preliminary results, they highlight that it is essential to monitor daytime sleepiness carefully, especially in adolescents with mobile phone addiction.

### Daytime sleepiness and depression as chain mediation between mobile phone addiction and suicidality

Our study adds to previous research showing that mobile phone addiction could affect daytime sleepiness which contributes to depressive symptoms, and subsequently increases the risk of suicide. This chain mediating effects highlights daytime sleepiness may be a precursor to depressive symptoms, especially among teenagers who are heavily exposed to mobile phone use. Our results were partly consistent with a previous Korean study indicating that insufficient sleep time and feeling sleepy during the day in adolescence was significantly associated with depression [102]. Therefore, monitoring sleep duration in adolescents and reducing daytime dozing may effectively reducing depressive symptoms, which in turn may lower the risk of suicide. The mechanism between daytime sleepiness and depressive symptoms may require more research to explore, such as longitudinal studies and a combination of biological and neuroscience research methods.

### Limitations, strengths, and future directions

One of our strengths is the use of follow-up design to explore how mobile phone addiction predicts later suicide risk in a representative adolescent sample. The current study added further and reliable evidence for the relationship between adolescent behavior addiction and suicide risk, and provided evidence and reference for further intervention. There were several limitations in the present study. First, the study sample was recruited in areas classified as moderate risk COVID-19 prevalence areas, which means that the results are not necessarily applicable to adolescents in other areas. Second, the psychological variables investigated in the present study were limited; therefore, future studies could include more variables such as sleep disorders, anxiety, interpersonal relationships, and acute stress. Third, as no participant information was gathered before the pandemic, the pre-COVID mobile phone use, daytime sleepiness, depression, and suicidality were unknown. Fourth, since this study uses a self-assessment questionnaire, the subjective deviation is inevitable. More reliable tools such as face-to-face interviews or objective indicators are needed in future research. Fifth, because depression is positively correlated with daytime sleepiness, it is difficult to explore the causal relationship between the two because it is cross-sectional data, and future studies need to explore the causal connection in a longitudinal design. Finally, as the period between the two waves was relatively short, longitudinal studies over more extended periods and different survey waves would provide a more transparent, long-term picture.

### Conclusion and implications

This study was one of the few studies that have examined the prospective associations between mobile phone addiction and suicidality in a short-term longitudinal study during COVID-19 in a large cohort of adolescents. The key findings were that mobile phone addiction during the COVID-19 quarantine period could, directly and indirectly, predict suicidality five months later when the pandemic was in remission. Depression and daytime sleepiness plays a mediating role in linking mobile phone addiction and suicidality. These findings confirmed the importance of long-term regular monitoring of suicide risk, depression, and daytime sleepiness in adolescents with mobile phone addiction. Long-term psychosocial care or support is needed for vulnerable adolescents with depression, and daytime sleepiness, as these could be warning signs for suicide risk detection, prevention, and intervention.

## Data Availability

Data could be obtained from the corresponding authors upon reasonable request.
